# Capacity building for health inequality monitoring in Indonesia: enhancing the equity orientation of country health information systems

**DOI:** 10.1080/16549716.2017.1419739

**Published:** 2018-03-23

**Authors:** Ahmad Reza Hosseinpoor, Devaki Nambiar, Jihane Tawilah, Anne Schlotheuber, Benedicte Briot, Massee Bateman, Tamzyn Davey, Nunik Kusumawardani, Theingi Myint, Mariet Tetty Nuryetty, Sabarinah Prasetyo, Suparmi, Rustini Floranita

**Affiliations:** a Department of Information, Evidence and Research, World Health Organization, Geneva, Switzerland; b The George Institute for Global Health, Delhi, India; c World Health Organization, Jakarta, Indonesia; d World Health Organization, New Delhi, India; e The US Agency for International Development, Jakarta, Indonesia; f Shool of Public Health, The University of Queensland, Brisbane, Australia; g Indonesia Agency for Health Research and Development (IAHRD), Jakarta, Indonesia; h Badan Pusat Statistik, BPS-Statistics, Jakarta, Indonesia; i Faculty of Public Health, Universitas Indonesia, Depok, Indonesia

**Keywords:** Monitoring Health Inequality in Indonesia, capacity building, Indonesia, health inequality monitoring, health equity, health information systems

## Abstract

**Background**: Inequalities in health represent a major problem in many countries, including Indonesia. Addressing health inequality is a central component of the Sustainable Development Goals and a priority of the World Health Organization (WHO). WHO provides technical support for health inequality monitoring among its member states. Following a capacity-building workshop in the WHO South-East Asia Region in 2014, Indonesia expressed interest in incorporating health-inequality monitoring into its national health information system.

**Objectives**: This article details the capacity-building process for national health inequality monitoring in Indonesia, discusses successes and challenges, and how this process may be adapted and implemented in other countries/settings.

**Methods**: We outline key capacity-building activities undertaken between April 2016 and December 2017 in Indonesia and present the four key outcomes of this process.

**Results**: The capacity-building process entailed a series of workshops, meetings, activities, and processes undertaken between April 2016 and December 2017. At each stage, a range of stakeholders with access to the relevant data and capacity for data analysis, interpretation and reporting was engaged with, under the stewardship of state agencies. Key steps to strengthening health inequality monitoring included capacity building in (1) identification of the health topics/areas of interest, (2) mapping data sources and identifying gaps, (3) conducting equity analyses using raw datasets, and (4) interpreting and reporting inequality results. As a result, Indonesia developed its first national report on the state of health inequality. A number of peer-reviewed manuscripts on various aspects of health inequality in Indonesia have also been developed.

**Conclusions**: The capacity-building process undertaken in Indonesia is designed to be adaptable to other contexts. Capacity building for health inequality monitoring among countries is a critical step for strengthening equity-oriented national health information systems and eventually tackling health inequities.

## Background

Inequalities in health outcomes, access/use of health services, and in health behaviors represent a recalcitrant problem in many countries, including Indonesia [1,2]. Addressing health inequality is a central component of the Sustainable Development Goals (SDG): the concept of ‘leaving no one behind’ is at the bedrock of the health-related SDG 3 and the corresponding Target 3.8 for Universal Health Coverage [3–6]. Linked to this is Target 17.18, which calls for the global community ‘by 2020, [to] enhance capacity-building support to developing countries to increase significantly the availability of high-quality, timely, and reliable data disaggregated by income, gender, age, race, ethnicity, migratory status, disability, geographic location and other characteristics relevant in national contexts’ [4].

**Figure 1.**
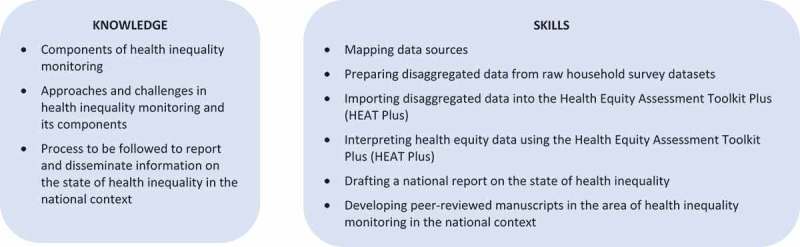
Specific capacities required for health inequality monitoring.

Source: Authors.

The mandate of the World Health Organization (WHO) includes providing technical support and building capacity for health inequality monitoring among member states [7–10]. Capacity building in health inequality monitoring at the country level includes development of both knowledge and skills, as outlined in Figure 1. Anticipating the capacity needed for health-inequality monitoring, WHO began developing a new approach involving ‘training of trainer’ workshops alongside health inequality monitoring workshops with member states across WHO regions [7]. The first workshop of this kind was held in Jaipur, India in 2014, where a number of representatives from different member states of the WHO South-East Asia Region, including Indonesia, participated.

The workshop was developed and facilitated by WHO, with support from trainers selected from reputed academic institutions in the region. Participants were exposed to key concepts and processes relevant to health inequality monitoring and gained direct experience working with disaggregated data using international household health surveys, interpreting summary measures of inequality, and using this information to highlight priorities for policymaking [2]. It was raised at the beginning of this meeting that while health inequality monitoring is desirable, very often, constraints of capacity, time, and the ready availability of tools to carry out monitoring preclude the introduction or institutionalization of this in country health systems. Jaipur solidified Indonesia’s commitment to health inequality monitoring through visualization of inequalities, and reporting of these inequalities through a state of health inequality report at the national level which could also serve as a baseline for equity-linked policymaking going forward. WHO was at that time developing a software application for assessing health inequalities. In 2016, this culminated in the creation of the Health Equity Assessment Toolkit [HEAT] [11], which contains the built-in WHO Health Equity Monitor database [12,13] and enables users to explore and compare within-country health inequalities using a variety of interactive tables and graphs. Alongside the capacity-building process in Indonesia, WHO developed an upload database edition of HEAT, i.e. HEAT Plus, which allows users to upload and work with their own data, drawing from a wide range of sources.

In Indonesia, a number of in-country stakeholders expressed an interested in using data from routine reports, household health surveys (such as the basic health research survey [RISKESDAS] and the national socio-economic survey [SUSENAS]), health facility surveys (such as the health facility research survey [RIFASKES]), as well as special disease surveys (such as the TB prevalence survey), which together cover a large range of health indicators disaggregated by a number of inequality dimensions [14]. Stakeholders included: the Indonesia Agency for Health Research and Development (IAHRD) (the coordinating institution); other departments across the Ministry of Health (Center of Data and Information, Family Health Directorate, International Cooperation Bureau, and Sustainable Development Goals Secretariat); Statistics Indonesia (Badan Pusat Statistik/BPS); the Centre for Health Economics and Policy Studies, and the Center for Health Research, Universitas Indonesia; the Center for Health Policy and Management, University of Gajah Mada; the United Nations Population Fund (UNFPA); the United Nations Children’s Fund (UNICEF); and the United States Agency for International Development (USAID), Indonesia. They committed to partnering with WHO and its trainer network to enhance capacity for monitoring health inequalities within the country.

The capacity-building process entailed a series of workshops, meetings, activities and processes undertaken between April 2016 and November 2017 (see Table 1). At each stage, a range of stakeholders with access to the relevant data and capacity for data analysis was engaged with, under the stewardship of state agencies. This included the Ministry of Health, universities, the statistics bureau, funders such as USAID, and other UN agencies, always with close interaction and support from across three levels of the WHO (at headquarters, in the region, and within Indonesia).

**Table 1.** Summary of key capacity-building activities and outputs for health inequality monitoring in Indonesia.

Title/type	Timeline	Objectives	Organization	Content	Outputs/action points
Training workshop using HEAT	April 2016	To build capacity in Indonesia in monitoring health inequality with a focus on interpreting data, and generating evidence-based inequality reports	WHO	Introduction of components of health inequality monitoring as well as the WHO Health Equity Assessment Tool (HEAT), definition of follow-up activities and expression of commitment from stakeholders	Outlines for (1) data source mapping exercise; (2) a state of health inequality report
Follow-up technical meeting	May 2016	To undertake data source mapping and create list of indicators for inclusion	IAHRD; with support from WHO; universities; USAID	Review of all relevant data sources; collation of indicators	Data source mapping and list of indicators; mapping of the team of each of the report chapters
					
Data preparation by technical groups	May–August 2016	Preparation of selected indicators for uploading to HEAT Plus (for statistical analysis)	IAHRD	Preparation of first draft of disaggregated data using the HEAT Plus format for all selected indicators	Disaggregated data using HEAT Plus format – first version
					
Follow-up technical meeting	August 2016	Refining the preparation of selected indicators for uploading to HEAT Plus	WHO	Preparation of second draft of disaggregated data using the HEAT Plus format for all selected indicators	Disaggregated data using HEAT Plus format – second version
					
Data preparation and import to HEAT Plus	September–November 2016	Uploading of selected indicators to HEAT Plus	IAHRD and WHO	Preparation of first draft of master data using the HEAT format for all selected indicators	Master data for HEAT Plus → first version
					
Training workshop using HEAT Plus	November 2016	To analyze and interpret the health equality data using HEAT Plus; refine outline of the state of health inequality report; test HEAT Plus	WHO	Beta testing and training using HEAT Plus Preparation of report chapters outline; Discussion on manuscript publication in an international journal special issue (Global Health Action); Agreement on timelines for report and publications;	First draft of national report of the state the health inequality; timeline for report and papers; draft of authors and co-authors of paper
					
Follow-up technical meeting	February 2017	To progress the chapters of the state of health inequality report	IAHRD	Draft report of each chapter	Revised report of each chapter→ second version
					
Data clinic and paper/report write-up workshop	April 2017	To discuss and progress both the report and the manuscripts for peer-reviewed publication – finalize analyses and complete advanced drafts	WHO	Data clinics to clarify data for inclusion in and statistical analysis for special issue articles; capacity building in writing for peer-reviewed publication and hands-on sessions regarding content for publication in the GHA special issue; content gathering for key sections of the report	Outlines of special issue articles; progress in statistical analysis for relevant special issue articles; additional content for key sections of the report
					
Report review meeting	September 2017	To review the national report on state of health inequality	WHO	Revised report	Providing feedback to finalize the technical editing of the report
					
					
Further development, review, finalization, and production of the report	April-November 2017	To fully develop the report	WHO	Compilation and technical editing of report text, development of graphics, review of and finalization of the report, and production of the publication including copy-editing, design, layout, and printing	Print and electronic report, interactive visuals
Launching the report	December 2017	To launch the report in a high-level national meeting	IAHRD	Report launch: presentation of findings and capacity-building process	Final report *State of health inequality: Indonesia*; planning to develop topic-specific policy briefs based on the published report
					
Submission of papers for special issue	July 2017­-February 2018	To document, for dissemination in the peer-reviewed literature, process, outputs, findings, and potential impact of the capacity building in inequality monitoring exercise	Authors, with technical support from WHO	Finalized manuscripts	Submission-ready manuscripts

WHO: World Health Organization; IAHRD: Indonesia Agency for Health Research and Development.

Workshops emphasized skill and capacity building, while technical meetings were focused on assessing progress, making key technical and logistical decisions, and course corrections to ongoing processes, as necessary.

The process was launched with a WHO-led training workshop in Jakarta in April 2016 to familiarize participants with health inequality monitoring and to introduce HEAT (http://www.who.int/gho/health_equity/assessment_toolkit/en/). At this workshop, participants from Indonesia expressed an interest in developing a state of health inequality report. This was followed by a data source mapping activity explained in detail elsewhere [14], and a technical meeting in Indonesia organized by the IAHRD to appraise the status and quality of data relevant for the proposed report. Between May and August of 2016, the IAHRD developed multiple versions of datasets drawing upon data from various data sources [14]. From September to November 2016, with close support from WHO technical experts and regional trainers, IAHRD led the production of the first version of an extended dataset for analysis. In November 2016, WHO Indonesia convened an in-country training workshop, facilitated by WHO technical experts and regional trainers to upload and analyse the first combined version of the Indonesian dataset. At this workshop, HEAT Plus was tested and used for analysis. The workshop served the dual purpose of allowing capacity to be built analysis and reporting inequality while also enabling feedback on user requirements and specifications regarding HEAT Plus, which could further inform improvements to the software. The outline of the *State of health inequality: Indonesia* report was also refined at this workshop, and attendees agreed to formalize the outputs of the collaborative inequality monitoring process, in the form of several manuscripts for peer-reviewed publication. Between November 2016 and April 2017, drafts of the *State of health inequality: Indonesia* report and various manuscripts for peer-reviewed publications underwent an iterative process of development. In April 2017, key stakeholders met in Yogyakarta to discuss and progress both the report and the manuscripts; WHO technical experts and regional trainers again provided analytical and write-up support. The *State of health inequality: Indonesia report* was further developed, reviewed, finalized and produced between April and November 2017 and launched in a high-level national meeting in December 2017 [15,16].

## Capacity-building outcomes

There were four key outcomes of the health inequality monitoring capacity-building exercise in Indonesia. First, over a series of workshops, technical meetings and opportunities for direct, group-based collaboration, capacity for monitoring health inequality – mainly statistical analysis, interpretation, and reporting of findings – was developed among a wide network of national stakeholders. Second, a report on the state of health inequality in Indonesia was produced, covering a wide range of health indicators disaggregated by a number inequality dimensions, such as education level, occupation, employment status, place of residence, age, sex, and subnational region. The third outcome, one that had been specifically sought by various stakeholders involved in the process, was the preparation of several peer-reviewed manuscripts included in this special issue of Global Health Action, which detail the process and outcomes of this exercise so as to help facilitate its adaptation/uptake in other country contexts. Finally, in order to embed this process within the national policymaking agenda, a launch event with attendance of all stakeholders, including technical experts and health policy makers, was convened in Jakarta in December 2017.

## Discussion

### Successes

The foremost reason for the progress and success of health inequality monitoring in Indonesia is the sustained political will across levels of administration and leadership in the country. Representation from Indonesia at training workshops and technical meetings was and remains strong and enthusiastic; teams are now able to support one another, reflect critically on the process, and advocate for its importance in health policymaking.

The effort involved one key government agency – IAHRD – which had a clear mandate and scope of responsibilities with respect to the various outputs envisioned under capacity building. This enabled role clarit*y*, complemented by a highly collaborative and well coordinated process of joint capacity building across stakeholders like other parts of the Ministry of Health, the national statistics bureau, universities, donors, WHO and other UN agencies playing critical roles throughout various phases (see Table 1). Another key success factor in the process in Indonesia was the concurrent development of specific software and technology for health inequality monitoring which enabled the use of local datasets. The existence of a technical handbook and easily accessible tools helped facilitate the application of the software to monitor health inequalities [7–9,11, 17]. At the heart of the collaborative process in Indonesia was the strong and ongoing support by the WHO country office, both at senior levels and by way of commitment and dedicated time of WHO staff, which helped maintain the momentum and relationships between stakeholders.

### Challenges, lessons learned, and moving forward

Certain phases of the capacity-building process introduced complications. For instance, access to raw datasets was not always forthcoming or immediate. In some cases, datasets had to be applied and paid for, while in other cases, access was not granted. For accurate health inequality monitoring, it was essential to have raw datasets, from which calculation of standard errors and 95% confidence intervals would be possible. Further, collaboration across various government agencies – specifically data management units – was essential to allow this sharing and use of data. While it was on the whole highly productive and desirable to have multiple analysts working on data preparation, this obviously introduced slight variations in process and in some cases, errors. To remedy this, greater synergy and interaction between data analysts (especially those from different agencies) is also highly desirable. Further, while there were reference tools to assist with the technical parameters of analysis, carrying out health inequality monitoring has many steps, particularly in the stage of data preparation. Participants in the April 2016 training workshop felt that more practical support was required as they progressed through the stages of preparing, analyzing, interpreting, and reporting data. A step-by-step manual was subsequently created based on the process followed in Indonesia and was made publicly available in July 2017 [18]. The fact that HEAT Plus was being developed in tandem with the capacity-building process in Indonesia was helpful in that the software could be immediately improved based on feedback and weaknesses identified by users. For example, a feature allowing users to highlight chosen data points in graphs was added based on feedback from this exercise. It must be noted that this resulted at times in delays while components and features were refined. As a result of the feedback process, however, HEAT Plus is now fully developed for application in other contexts.

The process of routine monitoring of inequalities described here must be supplemented by further capacity building in quantitative and qualitative research that can more deeply explore *why* inequalities exist and *how* they may be addressed [19,20,21]. In addition to reports specific to the state of inequality, health inequalities may also be incorporated into health sector progress reports, performance reports, and annual health statistical reports. This will allow health inequality data to be triangulated across sources for a deeper and richer understanding of a country’s situation in relation to health equity.

Inequalities in health services and health outcomes exist between and within provinces in Indonesia. In the context of the current decentralized government system in Indonesia, capacity building at the provincial level – where specific local factors and need by district are likely to be better understood – may help to reduce health service and health outcome inequalities at the national level.

Finally, to enable ongoing comprehensive national monitoring, dimensions of inequality need to be routinely included in surveys, civil registration, health facilities, and other relevant data sources. For this to happen, a consensus must be generated across agencies and arms of government to include priority dimensions of inequality (typically available dimensions like economic status and education, but also country-specific dimensions like subnational units, ethnicity, or migration status). This, of course, requires a longer and more elaborate process, one that will allow countries to progress in relation to the aforementioned Target 17.18 of the SDGs in addition to a number of other goals beyond health.

### Adaptation and implementation in other countries/settings

The process of capacity building for health inequality monitoring undertaken in Indonesia and outlined in this paper is designed to be adaptable to other contexts, focusing on national priorities and subnational programs with links to the broader global SDG agenda. The process is nonetheless reliant on the factors presented earlier: (1) strong political will; (2) having a key ‘champion’ agency; (3) role clarity; (4) demand-driven use of technology and tools; and (5) strong coordination and backstopping from WHO  and other relevant international technical agencies. We believe these elements are essential for capacity building in health inequality monitoring. Further, health inequality monitoring is a necessary but insufficient component of enhancing the equity orientation of health systems; this requires a range of other strategies and processes, which may also require the five aforementioned factors to be in place.

For most countries, a critical first step (see Table 1) is to carry out data source mapping [14], which is essential for identifying the availability of data for health inequality monitoring. This activity can be coordinated by one key agency in collaboration with all stakeholders who are in possession of relevant data sources and have the mandate for analysis, with support from WHO. In further efforts in Indonesia, as elsewhere, it would be important to engage with a broad range of stakeholders including Ministry of National Development and Planning, professional organizations, as well as community and civil society organizations engaged with the issue of health equity. This may place additional capacity-building demands on the nation, which the WHO and technical partners are in a position to support.

## Conclusion

This capacity-building process has shown that health information systems may be marshaled to redress health inequalities in a collaborative and mutually fruitful process of capacity building. There are certain key ingredients but also room for customization and adaptation. Our experience in Indonesia has shown that in as much as embedding health inequality monitoring in national health information systems aims to leave no one behind, the process itself can be one that progressively seeks to bring everyone along!

## Paper context

In the SDG era, there is a strong impetus to increase the equity orientation of national health systems. This article details the capacity-building process for national health inequality monitoring in Indonesia and discusses successes and challenges, and how this process may be adapted and implemented in other countries/settings. Drawing from this experience, similar capacity building with broad stakeholdership in other countries could greatly advance health equity, in service of national and global goals.
